# PHLPP1 phosphatase inhibition in hypothalamus restores insulin signaling and action and reduces adiposity in obese rats

**DOI:** 10.1186/1758-5996-7-S1-A131

**Published:** 2015-11-11

**Authors:** Bruna dos Santos Cardoso, Gisele Castro, Natália Ferreira Mendes, Paula Gabriele Fernandes Quaresma, Tamires Marques Zanotto, Mario Jose Abdalla Saad, Patrícia de Oliveira Prada

**Affiliations:** 1 UNICAMP, São Paulo, Brazil

## Background

Obesity results from imbalance between food intake and energy expenditure. The energy homeostasis is regulated by hypothalamic neurons that receive different neural, hormonal and metabolic signals. Insulin is one of the main hormones that regulate energy homeostasis and acts through a cascade of intracellular signaling that depends on the activation of several proteins, such as Akt. Our hypothesis is that PH domain and Leucine rich repeat Protein Phosphatase 1 (PHLPP1) inhibits Akt activity by dephosphorylating serine 473 residues, impairing the insulin action in the hypothalamus. However, PHLPP1 expression and the role played by it on the hypothalamus of diet induced obesity (DIO) animals are not fully understood.

## Objectives

To investigate PHLPP1 protein expression in the hypothalamus of DIO rats and to assess whether the PHLPP1 silencing improves insulin action and decreases body adiposity.

## Methodology

Eight-week-old male Wistar rats were distributed into two groups. One group was fed with standard diet and the other group with high-fat diet, during eight weeks. After this period, the hypothalamus of Chow and DIO animals were dissected for analysis of PHLPP1 protein expression. In another experiment, DIO animals were cannulated in the lateral ventricle and received intracerebroventricular (ICV) treatment to silence PHLPP1 expression, or its control, scramble (siRNA-SCR), during 7 days. Body weight and food intake were measured daily. On day 8, fasted animals received an insulin or saline ICV injection and their hypothalamus was extracted after 15 min to evaluate the PHLPP1 expression and insulin pathway proteins. Retroperitoneal and epididymal adipose depots were dissected and weighted.

## Results

Initially, DIO animals showed increased protein expression of PHLPP1 in hypothalamus compared to the Chow group. After 7 days of ICV treatment with siRNA-PHLPP1, DIO rats showed reduced expression of this phosphatase and led to greater weight loss and reduction in adiposity, epipydimal and retroperitoneal. Interestingly, hypothalamic PHLPP1 inhibition restored the phosphorylation of Akt in the hypothalamus. Conversely, it was not observed significant changes in food intake and fasting glucose.

## Conclusion

Hypothalamic PHLPP1 silencing in DIO animals restored, at least in part, the insulin signaling, promoting reduction in body weight and adiposity.

